# A Latent Class Analysis of Adverse Life Events for Kenyan Adolescents

**DOI:** 10.1007/s40653-023-00603-4

**Published:** 2023-12-23

**Authors:** Paulo Correia Ferrajão, Bárbara Tourais, Inês Faria, Joana Dias, Ask Elklit

**Affiliations:** 1 Faculdade de Ciências Sociais e Tecnologia, Universidade Europeia, Lisbon, Portugal; 2 National Center for Psychotraumatology, University of Southern Denmark, Odense, Denmark

**Keywords:** Adverse life Events, National Sample, PTSD, Kenya, Polyvictimization

## Abstract

Extant evidence indicates that exposure to adverse childhood experiences (ACE) tend to cluster among children and adolescents. Considering that adolescents from African countries present higher risk of being exposed to multiple ACE compared to other countries, the identification of victimization profiles in this population is clearly warranted. The aim of this study was to determine meaningful clusters of individuals with similar experiences of ACE in a sample of Kenyan adolescents. Latent class analysis (LCA) was conducted to identify latent classes of exposure to ACE. In addition, the relationships between the latent classes and gender, parental education, living arrangements and diagnosis of post-traumatic stress disorder (PTSD) were estimated. A three-class solution was found to be the best description of ACE, and the classes were labelled ‘‘Low Risk’’, ‘‘Intermediate Risk’’, and ‘‘High Risk’’. Compared with the Low-Risk class, the High-Risk class was found to be significantly more likely to have a diagnosis of PTSD and being a female may be an antecedent risk factor for high exposure to ACE. The Intermediate Risk class was significantly less likely to have parents with high school or college education. This paper indicates that Kenyan adolescents present higher risk of being exposed to multiple ACE and that trauma research may turn its focus on the individual as the unit of analysis rather than traumatic events.

The study of the impact of adverse childhood experiences (ACE) on adolescents’ mental health is crucial for both prevention policies and intervention in this population. However, there is a dearth of research on the experience of ACE in adolescents, particularly in the low- and lower-middle-income countries (LALMIC), such as Kenya. This is remarkably relevant considering extensive evidence on the association between ACE with mental health disorders, health-risk behaviors and suicidal attempts, and physical disorders in children and adolescents from African countries (Bayer et al., 2007; Harder et al., 2012; Karsberg & Elklit, 2012; Klasen et al., 2010; Okello et al., 2007). Therefore, the study of the prevalence of exposure to traumatic events in LALMIC is highly needed.

It is well recognized that adolescence is a developmental period with high risk of experiencing ACE (Breslau et al., 1996). Several studies observed that adolescents reported a high magnitude of exposure to multiple ACE (Dyregrov & Yule, 2006; Feeny et al., 2004; Finkelhor et al., 2007). Furthermore, adolescents exposed to one kind of ACE are more likely to being exposed to other forms of ACE, thereby increasing the risk of mental disorders (Contractor et al., 2018; Zerach & Elklit, 2020), namely posttraumatic stress disorder (PTSD) (Costello et al., 2002; Dyregrov & Yule, 2006).

## Exposure to Trauma among Adolescents in Kenya

Exposure to ACE has been proposed as a risk factor for mental disorders. This seems particularly noticeable in African countries, such as Kenya, where the prevalence of mental health disorders among adolescents is particularly high. A few studies conducted in samples of Kenyan youth found that 27-35% rural school youth met full criteria for PTSD (Karsberg & Elklit, 2012; Mbwayo et al., 2019) and one quarter of youths from Nairobi schools screened positive for PTSD (Harder et al., 2012).

The high prevalence of PTSD in African adolescents may be related to high exposure to multiple ACE in this population. Africa is a continent where millions of people, especially children, suffer from hunger, disease, sexual or physical abuse, and violence (Karsberg & Elklit, 2012; Njenga, 2002). In accordance, previous studies documented higher prevalence of exposure to ACE among children and adolescents from LALMIC, especially in African countries, compared to European, Asian, and North American adolescents (Betancourt et al., 2013; Karsberg & Elklit, 2012; Le et al., 2018; Swahn et al., 2012).

However, most studies have only examined the effects of singular ACE (e.g., sexual abuse) on adolescents’ mental health problems which precludes the understanding about the cumulative effects of multiple lifespan traumas (Contractor et al., 2018). The concept of ‘‘polyvictimization’’ was introduced to describe the exposure to multiple potentially traumatic events (PTE) in children and adolescents (Finkelhor et al., 2007). The study of the impact of exposure to multiple PTE derives from literature which has documented that many children and adolescents are not only exposed to a single traumatic event but rather experience on-going or multiple victimizations (Claussen & Crittenden, 1991).

In some cases, victimization seems to be more a “condition” than an “event” as portrayed in the early traumatic stress literature (Finkelhor et al., 2007). This is particularly notorious in African samples such as Kenyan adolescents (Betancourt et al., 2013; Karsberg & Elklit, 2012; Le et al., 2018). Many children and adolescents across Kenya witnessed or experienced sudden war-like violence within their community due to the contested presidential election in Kenya in December 2007. For example, in Nairobi it was experienced very high levels of violence for over a month including burning stores and homes, forced circumcision, rape, and murder. Therefore, it seems likely that many Kenyan adolescents were exposed to multiple ACE.

## Latent Class Analysis

The study of traumatization among adolescents requires a comprehensive approach to identify victimization profiles. This approach allows to grasp the interrelationships among victimizations and the effect of these interrelationships on mental health problems, as well as the identification of groups of children who present similar risks of victimization (Finkelhor et al., 2007). This perspective is supported by previous empirical evidence in which it has been found that victimizations tend to cluster among adults (Hope et al., 2001; Outlaw et al., 2002) and children (Nishina & Juvonen, 2005; Saunders, 2003). Therefore, the assessment of multiple types of victimization seems highly important to distinguish different subgroups of polyvictimized individuals.

The experience of polyvictimization has been conceptualized differently by several authors. The cumulative approach postulates that the exposure to various types of traumatic events, the dose-response effect of the number of traumas, is associated with poorer outcomes in terms of mental health (Appleyard et al., 2005; Contractor et al., 2018; Finkelhor et al., 2007). The hierarchical approach assumes that victimizations are not equivalent in their traumatic potential and different types of ACE have differential impact on individuals’ mental health (Briere et al., 2016; Campbell et al., 2016; Stempel et al., 2017). Finally, the categorial approach assumes that individuals who overcome the mean number of types of victimizations among all victimized children - identification of a threshold of number of different ACE – present a higher risk of developing mental health problems, that is, individuals below this threshold are not polyvictimized (Finkelhor et al., 2007).

To better understand individual and complex experiences of child victimization, several researchers have moved beyond variable-centered analytic methods, using person-centered analytic techniques, such as latent class analysis (LCA). The use of LCA allows to determine latent and meaningful subgroups of polyvictimized individuals by considering both the count and types of traumas in delineating and identifying polyvictimization classes (Nugent et al., 2012) and their association with physical and mental health correlates (Contractor et al., 2018). Therefore, it allows us to empirically identify patterns of ACE exposure that occur most frequently in each sample and classify individuals with similar characteristics of ACE exposure into classes, i.e., groups (Shin et al., 2018).

Several studies were conducted to identify clusters or latent classes of adolescents who have experienced similar patterns of ACE. In a meta-analytic review, Contractor et al. (2018) noticed that several studies found a best-fitting solution of three or four classes. The authors identified across all studies three main classes that shared similar characteristics: first, a latent class with lower endorsement of the evaluated traumas compared to other classes (“low-trauma class”); second, a class characterized by a higher endorsement of specific traumas (“specific-trauma-class”); and third, a high-trauma class characterized by high exposure to different traumas during different developmental periods. It was also found that individuals belonging to the high-trauma class reported greater mental health problems compared to those belonging to other classes (Barboza, 2018; Contractor et al., 2018; Debowska et al., 2017).

Meanwhile, most LCA studies in adolescents have been conducted among North American adolescents. For example, Nylund et al. (2007) conducted a LCA to identify subtypes of victimization among children. The authors found a three-class solution according to the degree of exposure to ACE: a class with high victimization, a “sometimes” victimized class, and a non-victimized class. Pears et al. (2008) used LCA to identify subgroups with different experiences of maltreatment in a sample of pre-school children, finding a four-class solution: a group who experienced supervisory neglect and emotional maltreatment; a group who experienced sexual abuse, emotional maltreatment, and neglect; a group who experienced physical abuse, emotional maltreatment, and neglect; and a group who experienced sexual abuse, physical abuse, emotional maltreatment, and neglect.

In European countries, Shevlin and Elklit (2008) found a four-class solution in a nationally representative sample of Danish adolescents: a low-risk class (only exposed to death of a family member and threat of physical attack); an intermediate risk class (low exposure to ACE but higher exposure compared to the low-risk group); a pregnancy/abortion class; and a high-risk class. Dunn et al. (2011) conducted a study in a nationally representative sample of British adolescents in which it was retrieved a four-class solution: a low adversity class; a class characterized by atypical parenting; a class characterized by loss of a family member, family discord, financial difficulties, maternal psychiatric illness, and atypical parenting; and a severe class that experienced child abuse.

## Latent Class Analysis in African Samples

There is a research gap in identifying the clustering of ACE in children from African countries, such as Kenya. In African samples, Clarke et al. (2016) explored the patterns of different types of violence suffered by Ugandan children. The authors found a three-class solution: a class of children who suffered physical violence by school staff (54.9% of the participants); a class of children exposed to physical, emotional, and sexual violence by peers (26.3% of the participants): and, a class of children exposed to emotional and physical violence by relatives, sexual and emotional abuse by girlfriends, boyfriends, and unrelated adults (18.8%) (Clarke et al., 2016). Ferrajão et al. (2022) conducted a latent class analysis to identity clusters of exposure to ACE in a sample of Ugandan adolescents. The authors identified a three-class solution: a group of adolescents with low probabilities of experiencing most of the adverse life events (“low risk class”); a group of adolescents with higher probability of being exposed to adverse life events compared to the “low risk” group (“intermediate risk class”); and, a group of adolescents with extremely high probabilities of having experienced all the adverse life events (“high risk class”) (Ferrajão et al., 2022).

In Kenyan samples, Miedema et al. (2023) conducted a LCA to assess classes of ACE in a nationally representative sample of male and female youth. The authors identified different solutions for males and females. In females, a five-class solution was found: a group exposed to sexual violence; a group exposed to household and community physical, emotional, and sexual violence; a group exposed to household and community physical violence; a group with low exposure to ACE; and a group exposed to emotional violence. In males, a four-class solution was found: a group exposed to household and community physical and emotional violence; a group with low exposure to ACE; a group exposed to household and community physical and sexual violence; and a group exposed to household and community physical violence.

## Purpose of the Study

Unfortunately, most of the prior studies on clustering of ACE only refer to North American and some European countries. Additionally, there is a lack of knowledge whether clusters of ACE may also be identified among the African children, such as Kenya. A few studies have been conducted on African samples to identify ACE clusters, focusing on the experience of violence but without analyzing the patterns of exposure to other ACE also very common in African adolescents, such as severe illness, death of someone close, and absence of a parent.

Gaining knowledge about the clustering of ACE in Kenyan adolescents is particularly relevant for prevention and treatment purposes. This is particularly relevant in a country like Kenya given that for many adolescents polyvictimization is not an exception, but a condition. Moreover, adolescents with similar exposure to ACE may benefit from similar types of treatment (Lanier et al., 2018).

The aim of this study was to determine if there are meaningful clusters of individuals with similar experiences of ACE in a sample of Kenyan adolescents based on the and by background variables (sex, living arrangements and parental education) and diagnosis of PTSD. Our research questions were: (a) To determine if there are meaningful clusters of individuals with similar experiences of adverse life events in a sample of Kenyan adolescents; (b) To analyze the relationship between latent classes and background variables (sex, living arrangements and parental education) with PTSD diagnosis. Findings would inform treatment interventions in Kenya and other African settings.

## Method

### Participants

Kenya is a country in East Africa that become a LALMIC in 2014, although poverty levels remain particularly high. Life expectancy at birth is around 57 years, and infant mortality rate and maternal mortality are high (Jenkins et al., 2015). Despite rapid urbanization, the agricultural sector is highly inefficient, and the food supply is vulnerable when catastrophic drought and floods occur. As mentioned above, Kenya has also suffered episodes of political turbulence and community violence.

The data for this study were collected from a questionnaire survey with a sample of 477 Kenyan school children aged 13 to 20 (*M* = 16.4; *SD* = 1.37). The sex distribution was 66.1% females and 33.9% males, and two participants did not disclose their sex. These two participants were removed from the subsequent analyses. Most of the participants lived with one of their parents (61.4%), 29.5% lived with both parents, and 9.1% had other arrangements. The differences between the parents’ education were not significant (fathers vs. mothers, respectively: no education 49.1% vs. 50.5%, primary school 17.8% vs. 22.6%, six years of education 16.8% vs. 18.7%, high school 9.4% vs. 5.7%, ‘‘college’’ 2.9% vs. 2.3%, and ‘‘university’’ (4.0% vs. 0.2%).

### Procedure

The primary aim to conduct the study was to gather information about trauma exposure and trauma reactions among Kenyan adolescents. This country was selected because it is a large, East African country that has experienced social unrest and civil war. The data were collected in 2012. Data collection was sponsored by the National Center of Psychotraumatology (Odense, Denmark). Three different boarding schools, one girls’ school and two boys’ schools were selected in Kenya. The boarding schools are a solution to an educational system with very few resources. In this situation, boarding schools are a solution for those parents who could afford to pay for it and have children who could meet the academic challenges of the secondary school. The teaching was supposedly of a higher quality than the village schools, which mainly covers primary education. And in many places, the boarding schools were the only school system for this age group. The schools were selected based on their characteristics that were viewed as typical. Because the national educational statistics were very sparse, we relied on the advice of a well-educated, licensed Danish psychologist who had been working in the country for 30 years as a school psychologist and served the authorities in building up psychology services in the schools.

The study was reviewed and approved by the institutional review board (IRB) of the Assistant District Education Officer of Laikipia North District, and by the headmasters of the three boarding schools. It was applied passive consent which is a usual procedure in most school studies in the LALMIC, i.e., the parents were informed about the study and had the right to refuse the participation of their child. In Kenya, the parents trust the school system and the teachers who are in parentis loco, i.e. they are granted the position to act in the best interests of their children. The participation was voluntary, and those accepting to participate gave their informed consent directly.

All the questionnaires were in English which is the official language of the country and the school system in Kenya. A pilot study with seven respondents at the age of 13–14 years was first conducted in the city of Nanyuki. From the pilot study it was obvious that the students had some language difficulties and that the questions to be answered by scales caused some trouble. It was observed a strong tendency that participants answered in the extremes in Likert scale-type questions. Therefore, the authors made a great effort of explaining the system of the scales and to create an atmosphere, where there were no such thing as ridiculous questions.

Participants filled out a questionnaire package containing questions concerning exposure to traumatic events along with the psychological impact of these events, and demographic variables. The questionnaires were filled out in the classroom. An average of 15–20 min was spent on introduction and explanation before the participants were asked to fill out the questionnaires. Participants spent approximately one and a half hour filling out the questionnaires. The researcher requested that the headmaster of all three schools would spare one or more teachers for each class so that they would be able to answer and explain the questions that the researcher was not able to and indeed the teachers were very helpful with this. The students were given pens and calculators as an appreciation of their help.

### Measures

The first part of the questionnaire contained questions about the participant. Specifically, details were recorded on gender, age, highest level of parental education and living arrangements.

The second part of the questionnaire contained 20 questions about traumatic events and life events they had experienced. This measure was developed by Bödvarsdóttir and Elklit (2007) who selected the list of events from scientific literature and clinical experience, and it is comprised of life-threatening experiences (e.g., rape) and stressful family conditions (e.g., neglect). Participants reported whether, or not, they had experienced each event. The sum of events that the adolescents were exposed was calculated by the number of different types of ACE that the participants reported that they had been directly exposed to. This measure has been widely applied cross-culturally including in samples of African adolescents (e.g., Ferrajão & Elklit, 2021). The reliability of the scale (Cronbach’s alpha = 0.84) was good.

The third part of the questionnaire included The Harvard Trauma Questionnaire Part IV (HTQ: Mollica et al., 1992). The HTQ assess both DSM-IV symptoms and culture specific symptoms associated with PTSD. This measure contains 30 items, 16 corresponding directly to DSM-IV-TR PTSD symptoms (American Psychiatric Association, 2000). The respondents were asked how much each symptom bothered them at the time when the event most distressing to them happened as *not at all* (1), *a little* (2), *quite a bit* (3), and *all the time* (4). The scale yields both a PTSD self-diagnosis according to DSM-IV criteria and a measure of PTSD symptom severity. The items are divided into three subscales that correspond to the three main symptom groups of PTSD: re-experiencing (“Recurrent thoughts or memories of the most harmful or terrifying events”), avoidance (“Avoiding activities that remind you of the traumatic or hurtful event”) and arousal (“Feeling jumpy, easily emotions”). Following the DSM-IV, the self-diagnosis of PTSD was made if participants reported at least one re-experiencing symptom, three avoidance symptoms and two arousal symptoms. A symptom was rated as present if the item corresponding to the symptom was scored 3 (‘‘quite a bit’’) or greater (Shevlin & Elklit, 2008). The cross-cultural validity of HTQ Part IV has been tested in at least two African settings; West Africa and South Africa (Kleijn et al., 2001; Renner et al., 2006). Cronbach alpha for the total score (0.87), re-experience scale (0.88), avoidance scale (0.90), and arousal scale (0.84) were good.

### Data Analysis

Missing data analysis were conducted before executing analyses. Proportion of missing values in the tested variables ranged from 0.2 to 7.5%. Missing data occurred because some participants did not respond to all items. A Little’s Missing Completely at Random (MCAR) was conducted which indicated that data were missing completely at random, χ^2^ (716) = 648.94, *p* = 0.97. Imputation of missing data was conducted through a maximum likelihood (ML) module. This procedure was adopted based on evidence of smaller degrees of bias in intermittent missing data (Shin, 2017). Specifically, each case of available data was used to compute ML estimates. The ML estimate of a parameter was the value of the parameter that was most likely to have resulted in the observed data (Bunting et al., 2002). The likelihood was computed separately for those cases with complete data on some variables and those with complete data.

LCA was conducted to identify the number and nature of subtypes of trauma exposure based on the absence or presence of direct exposure to each of the 20 traumatic events. LCA was performed using R Statistical Software (version 4.0; R Core Team, 2021). The fit of five models (two-class latent class model through to a six-class model) was assessed. The selection of the solution of latent classes with better fit was performed based on several statistical fit indices: Chi-square goodness of fit (χ^2^ goodness of fit), Akaike information criterion (AIC; Akaike, 1987), Bayesian information criterion (BIC; Schwarz, 1978), the Bootstrapped Lo-Mendell-Rubin’s adjusted likelihood ratio test (BLRT; Lo et al., 2001) and entropy measures (Ramaswamy et al., 1993).

A non-significant χ^2^ goodness of fit indicates acceptable model fit. The information statistics AIC and BIC are goodness-of-fit measures employed to compare competing models; lower observed values indicate better fit. When models with different numbers of latent classes are compared, the model with the lowest AIC and BIC is normally chosen as the best-fitting model. This is because a lower value of the information criterion suggests a better balance between model fit and parsimony (Lanza et al.,^2013^). The BLRT (Lo et al., 2001) statistic was used to compare models with differing numbers of latent classes. When it is obtained a non-significant value (*p* > 0.05), it indicates that the model with one less class should be accepted. Entropy (Ramaswamy et al., 1993) is a standardized measure of how accurately participants are classified, which ranges from 0 to 1 with higher values indicating better classification. Specifically, better classification indicates higher conditional probabilities in certain regions of the classification table (Wang & Bodner, 2007). In previous research, entropy values of 0.40, 0.60, and 0.80 respectively indicate poor, medium, and high classifications (Greenbaum et al., 2005; Muthén, 2004).

Selection of the best solution is also conducted based on other indicators helpful in identifying the optimal number of classes. The best-fitting model was also chosen based on parsimony of the model, average posterior probabilities (i.e., the diagonal values in the matrix of average latent class probabilities for most likely latent class membership closer to 1 and higher than 0.70) (Wang et al., 2012), profile size (i.e., models of small profile size less than 5% were rejected), and interpretability of estimated profiles, as supported by a substantive theory (Ferguson et al., 2020).

We then conducted a multinomial logistic regression to analyze the association between class membership (posterior probabilities from the model were used to assign each participant to their most likely class) with gender, highest level of parental education, living arrangements and PTSD diagnosis (clinical or subclinical). The odds ratios indicate the expected increase/decrease in the likelihood of scoring positively on a given variable compared with the reference, or control group.

## Results

### Prevalence of Exposure to Traumatic Events

As can be seen in Table 1, serious illness, and death of someone close were the most reported events, followed by witnessing other people injured or killed, came close to being injured or killed, absence of a parent, threats of violence and robbery/theft. Least prevalent events were miscellaneous events, rape, pregnancy/abortion, attempted suicide, and sexual abuse.

**Table 1:** Frequency of Direct Expose to Traumatic Life Events

	Female (n = 314)	Male (n = 161)	Total (N = 475)
	Count (%)	Count (%)	Count (%)
Traffic accident	72 (22.9%)	19 (11.8%)	91 (19.2%)
Other serious accidents	75 (23.9%)	24 (14.9%)	99 (20.8%)
Physical assault	75 (23.9%)	32 (19.9%)	107 (22.5%)
Rape	36 (11.5%)	11 (6.8%)	47 (9.9%)
Witnessed other people injured or killed	144 (45.9%)	39 (24.2%)	183 (38.5%)
Came close to being injured or killed	137 (43.6%)	42 (26.1%)	179 (37.7%)
Threats of violence	112 (35.7%)	50 (31.1%)	162 (34.1%)
Near-drowning	88 (27.7%)	23 (14.3%)	111 (23.4%)
Attempted suicide	55 (17.5%)	18 (11.2%)	73 (15.3%)
Robbery/theft	114 (36.3%)	48 (29.8%)	162 (34.0%)
Pregnancy/abortion	49 (15.6%)	17 (10.6%)	66 (13.8%)
Serious illness	181 (57.6%)	80 (49.7%)	261 (54.9%)
Death of someone close	174 (55.4%)	84 (52.2%)	258 (54.1%)
Divorce	101 (32.2%)	39 (24.2%)	140 (29.4%)
Sexual abuse	75 (23.9%)	20 (12.4%)	95 (19.9%)
Physical abuse	96 (30.6%)	36 (22.4%)	132 (27.8%)
Severe childhood neglect	97 (30.9%)	24 (14.9%)	121 (25.4%)
Humiliation or persecution (bullying)	108 (34.4%)	45 (28.0%)	153 (32.1%)
Absence of a parent	122 (38.9%)	55 (34.2%)	177 (37.3%)
Miscellaneous	35 (11.1%)	7 (4.3%)	42 (8.8%)

The average number of direct events per participants was 4.3 (10.5% did not report at least one traumatic event, 9.0% experienced one event, 10.3% experienced two events, 8.0% experienced three events, 8.2% reported four events, and 54.1% reported five events or more). The prevalence rate of PTSD, estimated from the total sample of 477 participants, was 31.0%.

As can be seen in Table 2, both the three-class solution and the four-class solution were the best-fitting models. The non-significant χ2 goodness of fit indicated acceptable model fit for both solutions. The four-class solution had lowest AIC, a significant BLRT, and a medium to high entropy. The three-class solution had lowest BIC, a significant BLRT, and high entropy which was the highest among all models. The selection of the best solution was conducted based on parsimony, average of posterior probabilities, profile size, and interpretability of estimated profiles as supported by theory. The three-class solution was preferred based on parsimony, higher average posterior probabilities, and it was the solution, apart from the two-class solution, with the highest value for the smallest group proportion. Moreover, the three-class solution was supported by previous LCA studies among adolescents (Barboza, 2018; Contractor et al., 2018; Debowska et al., 2017), namely among African adolescents (Clarke et al., 2016; Ferrajão et al., 2022), which also found a three-class solution.

**Table 2:** Fit Indices for the Latent Class Analysis of the Direct Traumas

Model	Log likelihood	χ^2^ goodness of fit	AIC	BIC	SSABIC	BLRT	Entropy	Smallest group proportion
2 classes (df) *p*	-4738	2650092.82 (436) 0.91	9558	9729	7197	277 0.001	0.69	47.7
3 classes (df) *p*	-4605	1702640.49 (415) 0.98	9335	9593	6932	78 0.01	0.86	13.6
4 classes (df) *p*	-4569	2763729.46 (394) 1.0	9305	9651	6860	66 0.01	0.77	7.8
5 classes (df) *p*	-4538	2295145.96 (373) 1.0	9285	9718	6798	36 0.16	0.69	6.5
6 classes (df) *p*	-4508	1775597.59 (352) 1.0	9265	9786	6737	31 0.79	0.68	3.5

*Note*. AIC = Akaike information criterion; BIC = Bayesian information criterion; BLRT = Bootstrapped Lo-Mendell-Rubin’s adjusted likelihood ratio

**Fig. 1:**
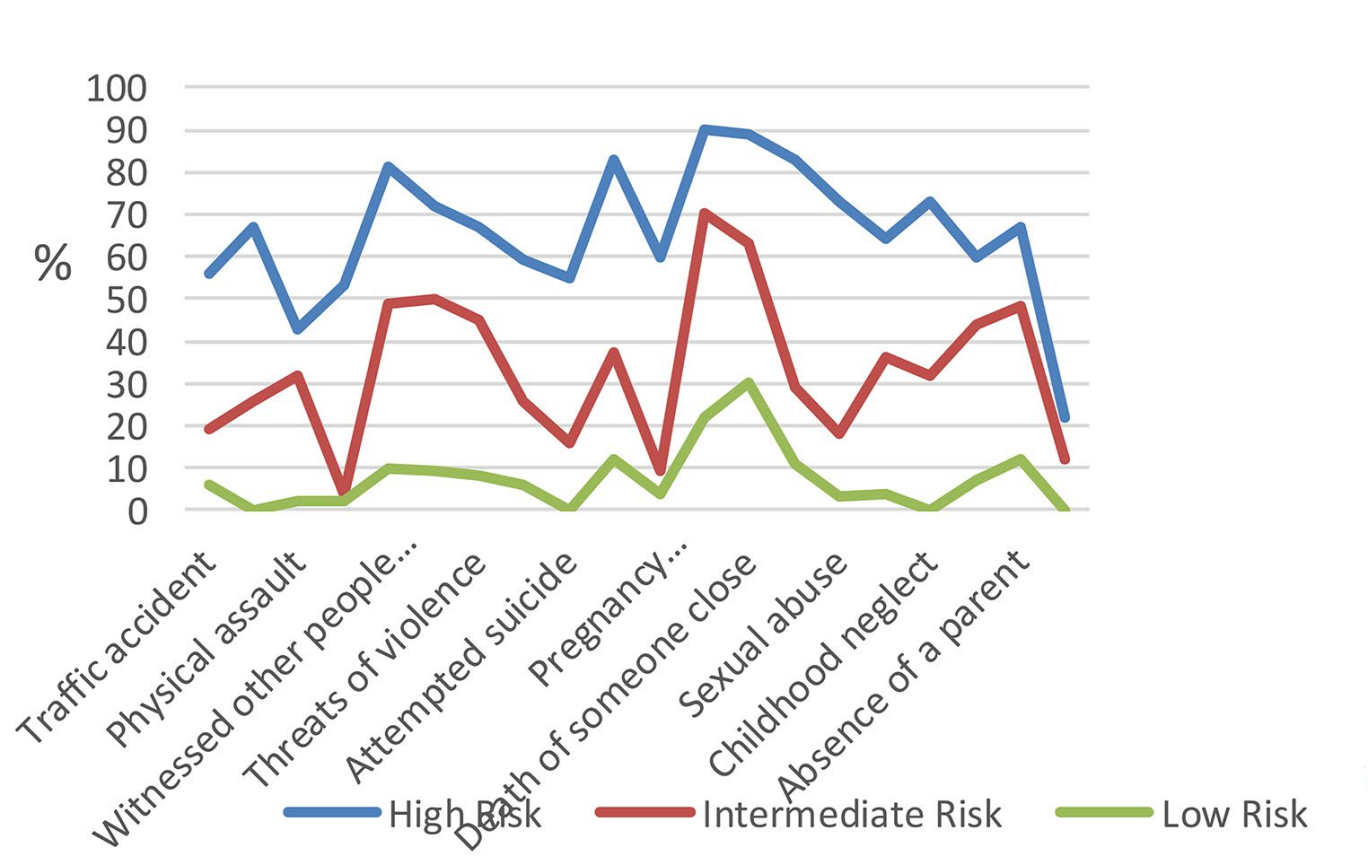
Estimated Class Conditional Probabilities in Latent Classes for Low, Intermediate and High Risk Groups. *Note*: Estimated class conditional probabilities of exposure to each of the adverse childhood experiences for the low, intermediate and high-risk groups. The green line represents the estimated class conditional probabilities for the high-risk group. The pink line represents the estimated class conditional probabilities for the intermediate-risk group. The gray line represents the estimated class conditional probabilities for the low-risk group

Both Table 3; Fig. 1 present the estimated class conditional probabilities in latent classes for low, intermediate, and high-risk groups.

**Table 3:** Estimated Class Conditional Probability

	Class 1: High Risk	Class 2: Intermediate Risk	Class 3: Low Risk
Traffic accident	0.56	0.19	0.06
Other serious accidents	0.67	0.26	0.00
Physical assault	0.43	0.32	0.02
Rape	0.53	0.04	0.02
Witnessed other people injured or killed	0.81	0.49	0.10
Came close to being injured or killed	0.72	0.50	0.09
Threats of violence	0.67	0.45	0.08
Near-drowning	0.59	0.26	0.06
Attempted suicide	0.55	0.16	0.00
Robbery/theft	0.83	0.37	0.12
Pregnancy/abortion	0.60	0.09	0.04
Serious illness	0.90	0.70	0.22
Death of someone close	0.89	0.63	0.30
Divorce	0.83	0.29	0.11
Sexual abuse	0.73	0.18	0.03
Physical abuse	0.64	0.36	0.04
Severe childhood neglect	0.73	0.32	0.00
Humiliation or persecution (bullying)	0.60	0.44	0.07
Absence of a parent	0.67	0.48	0.12
Miscellaneous	0.22	0.12	0.00

Class 3 included 184 participants (38.6%) and was characterized by very low probabilities of experiencing most of the adverse life events. The probabilities of experiencing the death of someone close (0.30) and serious illness (0.22) were relatively high followed by robbery/theft (0.12). This class was regarded as the baseline, or normative, group that included adolescents who are likely to experience few adverse life events. This class was labelled the ‘‘Low Risk’’ group. Class 2 had the highest proportion of participants (*n* = 228, 47.8%) and was characterized by higher probability of being exposed to adverse life events compared to Class 3. The probabilities of experiencing a serious illness (0.70), the death of someone close (0.63), came close to being injured or killed (0.50), witnessing other people being injured or killed (0.49), absence of a parent (0.48), and humiliation or persecution (0.44) were extremely high. This group has a similar pattern of probabilities to the normative group in terms of the low probabilities associated with some adverse events such as rape and pregnancy/abortion whereas for the remaining events their probabilities were higher. This class was labelled the ‘‘Intermediate Risk’’ group. Finally, Class 1 was the smallest class (*n* = 65, 13.6%) and was characterized by extremely high probabilities of having experienced all the adverse life events. This class was labelled the ‘‘High Risk’’ group. This class was also characterized by participants having a much higher probability of experiencing a serious illness (0.90), death of someone close (0.89), divorce (0.83), robbery/theft (0.83), witnessing other people injured or killed (0.81), sexual abuse (0.73), childhood neglect (0.73), and come close to being injured or killed (0.72).

Next, a multinomial logistic regression was conducted in which participants were allocated to a class based on their highest probability of membership to a class from the three-class solution. Class membership was introduced in the multinomial logistic regression model as a dependent variable. Variables representing gender, highest level of parental (father and mother) education (no education, primary, high school, college, or university), living arrangements (child lives with both parents or not) and PTSD diagnosis (present or not present) were entered as predictors. The Low-Risk group, acted as the reference group. Table 4 shows the likelihood ratio tests for the multinomial logistic regression.

**Table 4:** Results of the Multinomial Logistic Regression Predicting Latent Class Membership

Class 1					
Effect	-2 Log likelihood	Chi-square	df	*p*	OR (95% CI)
Intercept	84.50	0.00	0		
Gender	95.98	11.47		< 001	-3.46 (-4.06; -2.86)
PTSD	94.55	10.05	1	0.002	2.97 (2.32; 3.62)
Father education	85.24	0.73	4	0.95	-
Mother education	85.59	1.08	4	0.78	-
Living arrangements	86.03	1.53	1	0.22	-
Class 2					
Effect	-2 Log likelihood	Chi-square	df	*p*	OR (95% CI)
Intercept	147.63	0.00	0		
Gender	153.45	5.82	1	0.02	-1.87 (-2.31; -1.43)
PTSD	155.57	7.94	1	0.01	2.05 (1.59; 2.51)
Father education	166.30	18.67	4	< 0.001	-1.30 (-1.58; -1.02)
Mother education	149.69	2.06	4	0.72	-
Living arrangements	148.70	1.07	1	0.30	-
Class 3					
Effect	-2 Log likelihood	Chi-square	df	*p*	OR (95% CI)
Intercept	153.51	0.00	0		
Gender	164.33	10.82	1	< 0.001	2.13 (1.71; 2.55)
PTSD	166.37	12.86	1<	< 0.001	-2.24 (-2.68; -1.80)
Father education	169.11	15.61	4	0.004	1.25 (1.04; 1.46)
Mother education	155.00	1.49	4	0.83	-
Living arrangements	154.99	1.49	1	0.22	-
Predictor		Class 1: High Risk		Class 2: Intermediate Risk	
		OR (95% CI)		OR (95% CI)	
Gender (male)		-0.38 (-0.19; -0.77)		1.72 (1.06; 2.79)	
*p*		0.05		0.05	
PTSD (present)		2.79 (1.45; 5.34)		1.96 (1.22; 3.15)	
*p*		0.01		0.01	
Living arrangements (one parent or other arrangement)		0.67 (0.38; 1.20)		0.62 (0.37; 1.04)	
*p*		0.18		0.07	
Father education (no education)		-0.53 (-0.05; -5.73)		-0.70 (-0.20; -2.45)	
*p*		0.60		0.58	
Father education (primary)		-0.62 (-0.06; -6.73)		-0.58 (-0.17; -2.03)	
*p*		0.69		0.40	
Father education (high school)		-0.55 (-0.05; -6.49)		-0.18 (-0.05; -0.66)	
*p*		0.63		0.05	
Father education (college)		0.27 (-0.02; -4.48)		-0.10 (-0.02; -0.65)	
*p*		0.36		0.02	
Mother education (no education)		-0.18 (-0.14; -1.33)		-0.24 (-0.49; 1.23)	
*p*		0.88		0.11	
Mother education (primary)		-0.34 (-0.30; -0.38)		-0.31 (-0.06; -1.52)	
*p*		0.73		0.19	
Mother education (high school)		-1.01 (-0.47; -2.22)		-0.35 (-0.07; -1.85)	
*p*		0.75		0.23	
Mother education (college)		-1.07 (-0.31; -3.14)		-0.16 (-0.11; -0.38)	
*p*		0.66		0.79	

As can be seen in Table 4. the effects for the PTSD and gender were statistically significant for both the High-Risk and the Intermediate Risk groups, and father education was statistically significant for the Intermediate Risk group. Regarding the odds ratios associated with each predictor, it can be observed that, compared with the Low-risk group (Class 3), participants in the High-risk group (Class 1) are over three times more likely to have a diagnosis of PTSD (OR = 2.76, *p* < 0.01) and nearly half times more likely of being girls (OR=-0.38, *p* < 0.01). Finally, the Intermediate Risk group (Class 2) are over two times more likely to have a diagnosis of PTSD (OR = 1.96, *p* < 0.01) and nearly two times more likely of being males (OR = 1.72, *p* < 0.05), and one fifth times less likely to have parents with high school education (OR=-0.18, *p* < 0.01 and college education (OR=-1.60, *p* < 0.01).

## Discussion

To the best of our knowledge, this is the first study that was conducted to identify and distinguish different subgroups of individuals with similar experiences of adverse life events in a sample of Kenyan adolescents. The findings of the current study indicate that Kenyan adolescents show a high-risk of being exposed to multiple adverse life events. It was observed that having a serious illness, death of someone close, witnessing other people injured or killed, to be close of being injured or killed, and absence of a parent were the most reported events. Conversely, rape, pregnancy or abortion, attempted suicide, traffic accident, and sexual abuse were the least commonly reported events. The context of widespread economic precarity, hunger and high-risk of disease, and generalized community violence in Kenya are factors that may explain why exposure to adverse life events do not occur in isolation among Kenyan adolescents (Tsehay et al., 2020).

LCA was conducted to identify classes of adolescents with similar co-occurrence of adverse life events. A three-class solution was retrieved and considered the best-fitting model to the data. The LCA solution found in the current study shares similar characteristics with main classes identified in previous meta-analytic studies (Contractor et al., 2018) and studies with adolescents from the USA (Nylund et al., 2007) and other African samples (Ferrajão et al., 2022).

Specifically, Class 3 was labelled the low-risk group and was characterized by low probabilities of experiencing most of the adverse life events outside death of someone close and serious illness. It is recognized that many African countries, such as Kenya, are characterized by harsh conditions in which children and adolescents often suffer from hunger and disease (Karsberg & Elklit, 2012). Under such circumstances, some adverse life events such as death of someone close and suffering a serious illness are so common that probably are not appraised as excessively traumatic by these adolescents (Ferrajão et al., 2022; Njenga, 2002).

Class 2 was labelled the intermediate-risk group which presented higher probability of exposure to adverse life events compared to Class 3. This group had some similarities with the low-risk group because both groups showed low probability of experiencing serious ACE such as rape and pregnancy or abortion. However, this group of adolescents had a higher probability of co-occurrence of several serious traumatic events compared to the low-risk group, such as serious illness, death of someone close, came close to being injured or killed, witnessing other people being injured or killed, absence of a parent, and humiliation or persecution. The probability of being exposed to those events was quite pronounced. This group showed a pattern of ACE exposure as identified in previous studies (Shevlin & Elklit, 2008), including in samples of African adolescents (Ferrajão et al., 2022). The higher risk of exposure to ACE of this group compared to the low-risk group may be associated with the relatively high probability of absence of a parent which is a vulnerability factor for heightened confrontation with multiple ACE (Karsberg & Elklit, 2012; Le et al., 2018).

Finally, class 1 was labelled the high-risk group and members of this class showed extremely high probabilities of experiencing all the adverse life events assessed in the current study. Members of this group had a significantly higher probability of experiencing major traumatic events such as witnessing other people injured or killed, sexual abuse, childhood neglect, and come close to being injured or killed. The current findings indicate that this group of adolescents clearly presents a higher risk of chronic polyvictimization also observed in previous studies in samples of African adolescents (Clarke et al., 2016; Ferrajão et al., 2022; Miedema et al., 2023). Probably, the socioeconomical context lived in Kenya, characterized by hunger, disease, community violence, and experience of early traumas makes the experience of psychological trauma so common that it is viewed as an ordinary condition for some Kenyan adolescents (Karsberg & Elklit, 2012; Njenga, 2002).

The current findings are in accordance with previous theoretical background on higher probability of exposure to different types of ACE in African children and adolescents, namely in Kenya (Karsberg & Elklit, 2012; Le et al., 2018). It was also observed that the death of someone close and serious illness were events experienced by nearly all the participants which makes them a regular but not a traumatic event (Finkelhor et al., 2007). Classes 1 and 2 include groups of adolescents in which ACE tend to cluster according to different types of victimizations and number of types of victimizations (Briere et al., 2016; Finkelhor et al., 2007). The profile of class 1, which is characterized by high probability of being exposed to serious traumatic events (e.g., witnessing other people being injured or killed, sexual abuse) and other less serious ACE (e.g., death of someone close), indicates that harsh traumatic events often co-occur with other less severe traumatic adverse life events among Kenyan adolescents (Barboza, 2018; Debowska et al., 2017).

A multinomial logistic regression was conducted to evaluate the association between class membership and gender, highest level of parental education, living arrangements and PTSD diagnosis. Results showed that class 2 was significantly different from class 3 in terms of the probabilities associated with gender, PTSD and parental education, and class 1 was significantly different from class 3 in terms of the probabilities associated with gender and PTSD. Specifically, it can be proposed that having a parent with high school or college education is a protective factor against the likelihood of being exposed to a moderate number of traumatic events (class 2). It can also be proposed that adolescents with parents with a higher education level belong to a higher socioeconomic status which is a protective factor against exposure to ACE (Anderson et al., 2022).

Moreover, it seems that being a female may be an antecedent risk factor for high exposure to traumatic events (class 1) which significantly increases the likelihood of developing PTSD. These results were also found in previous studies among other African samples regarding higher risk of females in developing PTSD compared to males (Asnakew et al., 2021; Clarke et al., 2016; Jenkins et al., 2015). Higher risk of PTSD in females may be related to higher likelihood of suffering from childhood sexual abuse and sexual assault compared to males Asnakew et al., 2021; Rugema et al., 2015).

The current results are also in accordance with previous findings that underscored higher vulnerability of developing PTSD in adolescent at high-risk of exposure to ACE compared to those belonging to other classes with lower exposure to ACE (Costello et al., 2002; Dyregrov & Yule, 2006). The higher vulnerability of this group to the development of PTSD seems to be linked to a dose-response effect of the number of traumas (Contractor et al., 2018), but also by higher probability of being exposed to serious traumatic events such as sexual abuse, physical abuse and childhood neglect observed in this group (Campbell et al., 2016). Testing these causal hypotheses requires a study with a prospective design.

The current study has some limitations. Fist, experience of ACE was retrospectively assessed through a self-report measure which can suffer from memory and selectivity bias among respondents. Furthermore, self-reporting may result in under- or over- estimating experiences, particularly in the context of early adversity. Therefore, it is likely that different classes retrieved in this study may reflect differences in the types of adverse experiences, but also reflect differences in who reported those experiences. Future research would benefit from collecting information on multiple reporters for each ACE for classifying children and adolescents who experience co-occurring adversities. Second, experience of ACE was assessed using a dichotomous measure that may not adequately represent children’s experiences, as the severity of the experiences may differ between children. Future studies should measure the severity of adversity and how frequently an event occurred rather than using dichotomous indicators of adversity (Debowska & Boduszek, 2017). Third, PTSD was assessed using a self-report measure rather than structured interview. Despite a conservative approach adopted to positive PTSD diagnosis, there may have been an overestimation of the prevalence of PTSD. Fourth, PTSD diagnosis was not assessed using DSM-5 criteria. Future studies should be conducted using measures of DSM-5 criteria. Furthermore, the reported profiles of victimization should be analyzed in future studies among adolescents from other African countries. Fifth, information about parental occupation and family income was not collected. Future studies should collect such information and analyze these variables as predictors of class membership. Sixth, the data were collected in 2012. Thus, there may have been changes in the level of exposure to ACE among adolescents associated with temporal changes in the context of violence and adversity in Kenya.

Despite these limitations, the current findings bring some contributions to social welfare politics and clinical practice in this population. Considering extended evidence on high exposure to ACE among children and adolescents from African countries such as Kenya, the identification of subgroups of polyvictimized individuals using LCA is crucial for implementing strategies of prevention and intervention tailored for specific groups of individuals. Moreover, the combination of profiles of victimization with risk and resilience factors (personal and/or environmental) associated with specific profiles allows for a comprehensive approach of the adolescent before intervention. Consequently, in a context where resource scarcity prevails, the allocation of resources to the specific needs of adolescents and their environment is optimized. In addition, the current results indicate that adolescents who report serious traumatic events, such as sexual abuse and childhood neglect, were also likely to have experienced other less traumatic adverse life events such as illness and accidents.
